# Correction: Polling India via regression and post-stratification of non-probability online samples

**DOI:** 10.1371/journal.pone.0315139

**Published:** 2024-12-03

**Authors:** Roberto Cerina, Raymond Duch

The images for Figs 1 to 5 are incorrectly switched. The image that appears as Fig 1 should be Fig 4, the image that appears as Fig 2 should be Fig 5, the image that appears as Fig 3 should be Fig 1, the image that appears as Fig 4 should be Fig 3 and the image that appears as Fig 5 should be Fig 2. The figure captions appear in the correct order.

**Fig 1:**
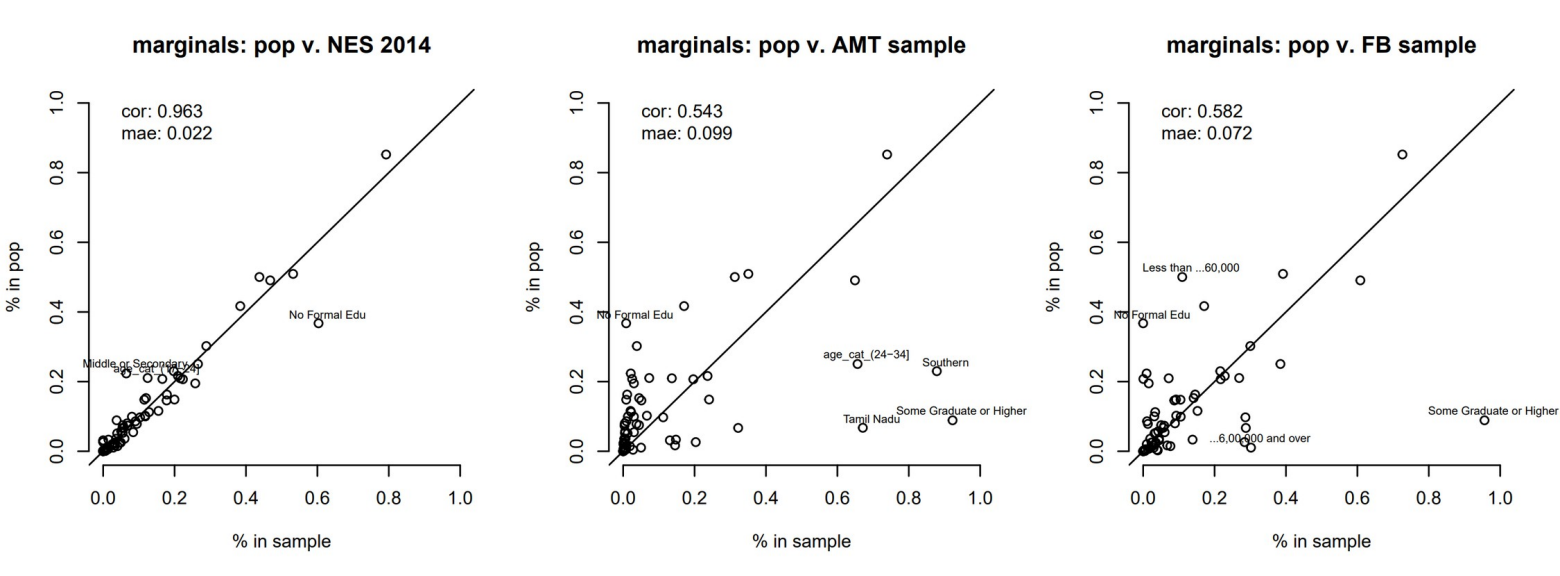
Summary plot assessing the degree of bias, when compared to the estimated stratification frame, across a number of marginal distributions, in each of the three samples used in the analysis. The most severe discrepancies are highlighted. Each dot represents the % of an attribute, such as education level, caste or income, in the population as a whole and in the sample at hand. If the sample at hand is perfectly representative of the population of interest, the dots should lie on the *y* = *x* line.

**Fig 2:**
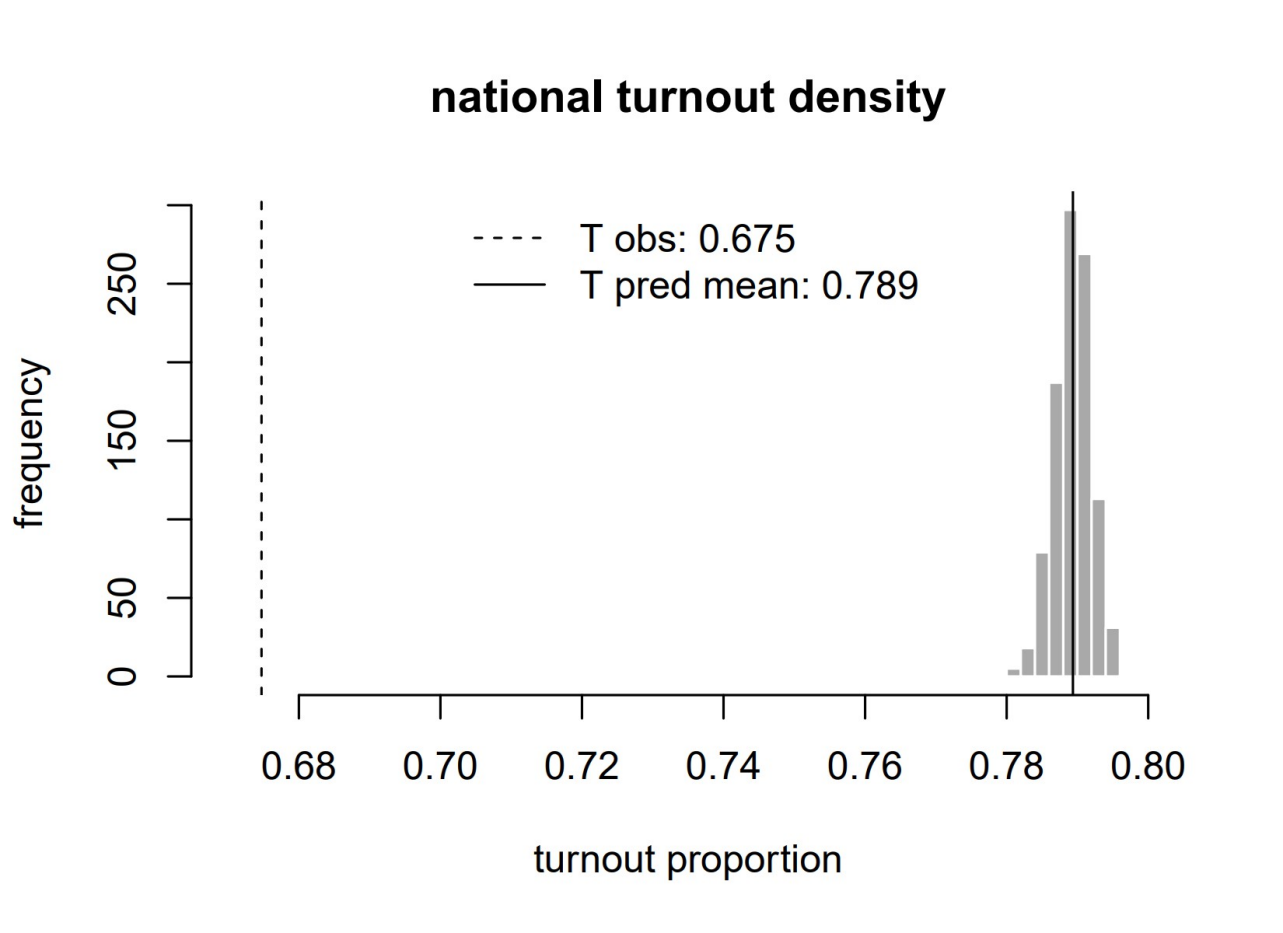
Predicted distribution of turnout at the national level.

**Fig 3:**
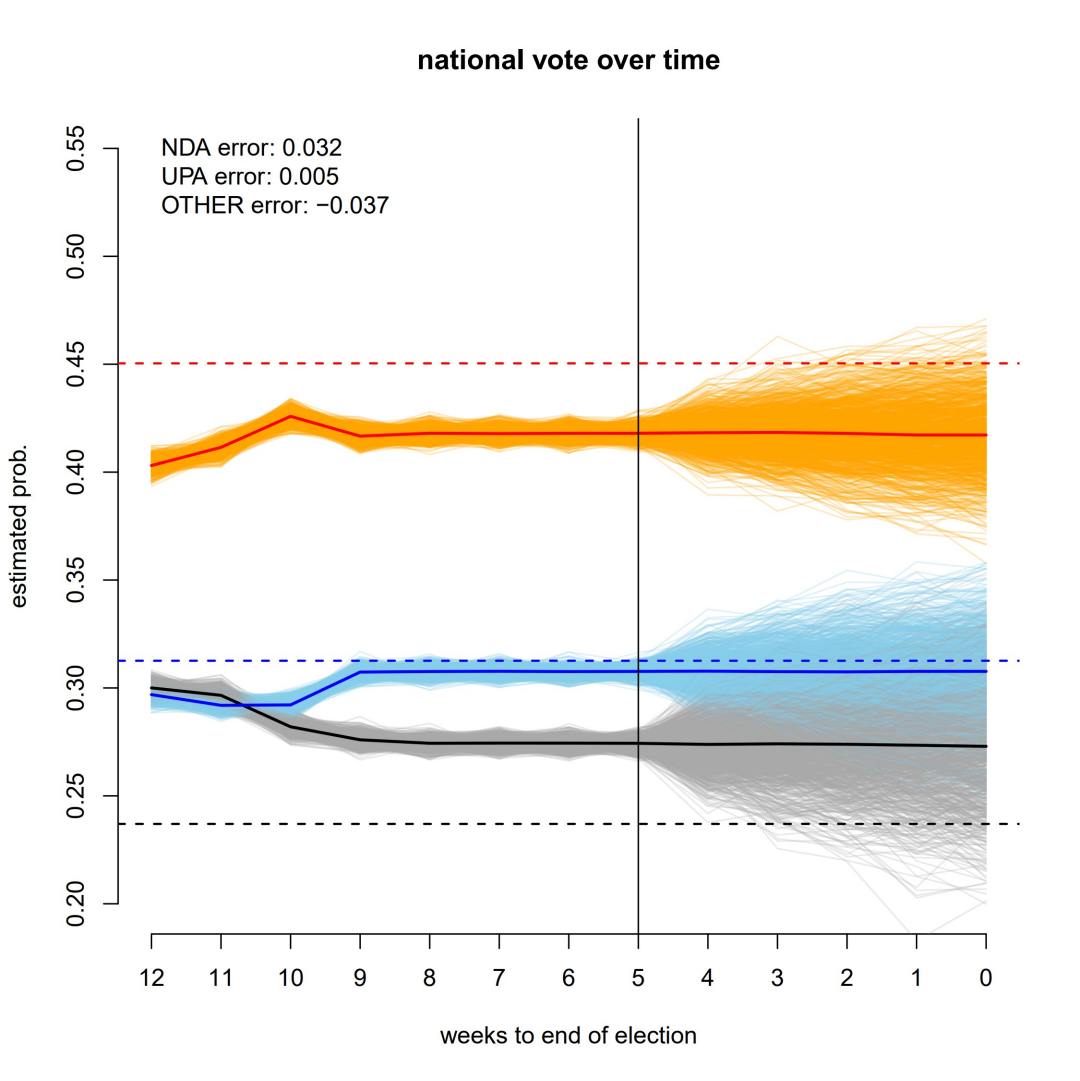
National vote share for the three major alliances over the course of the campaign; monitoring stops before the beginning of voting, and vote-share after this point.

**Fig 4:**
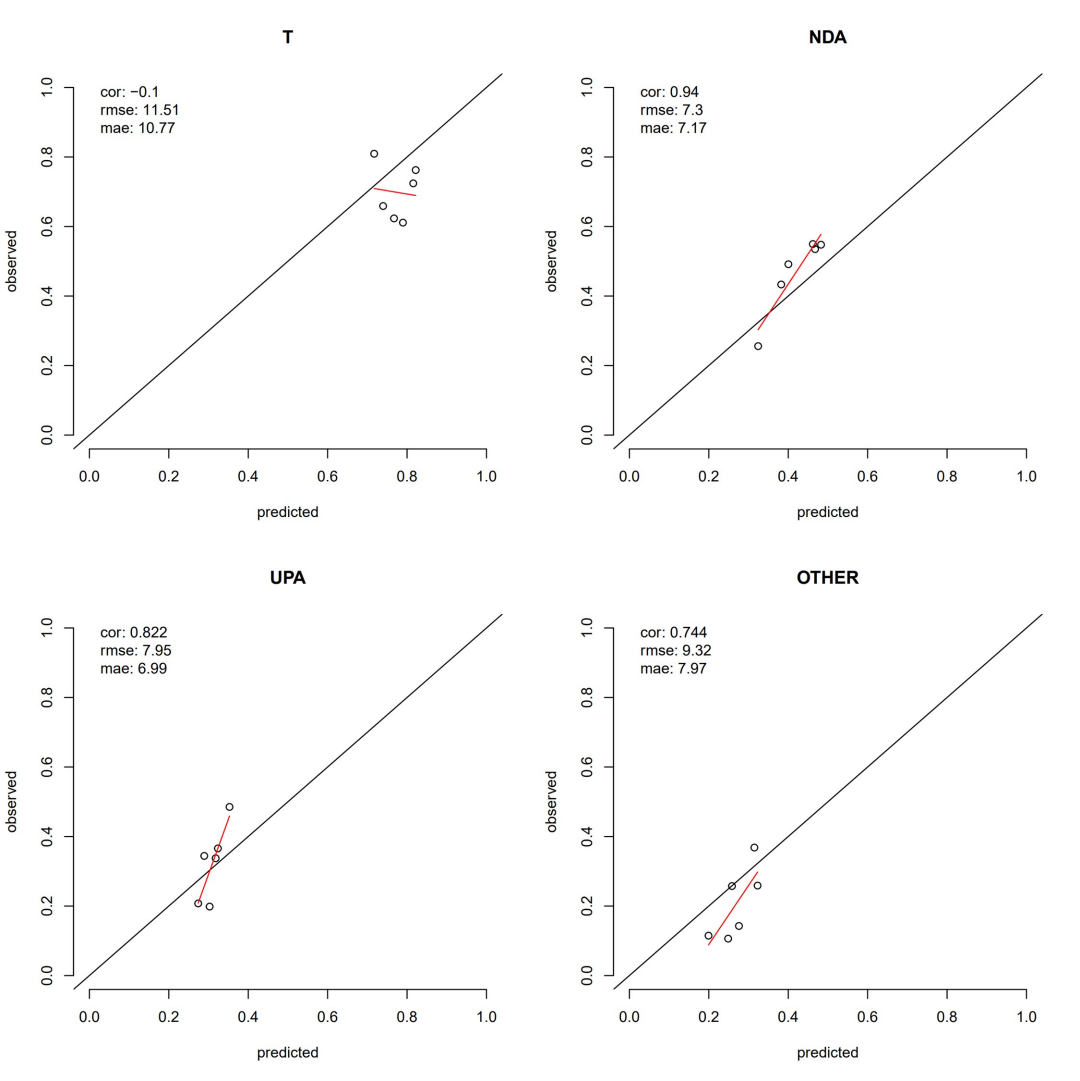
Zone-level predictions v. observed zone-level 2019 behaviour for Turnout share (top-left), and Alliance vote-share (NDA top-right, UPA bottom-left, Other bottom-right).

**Fig 5:**
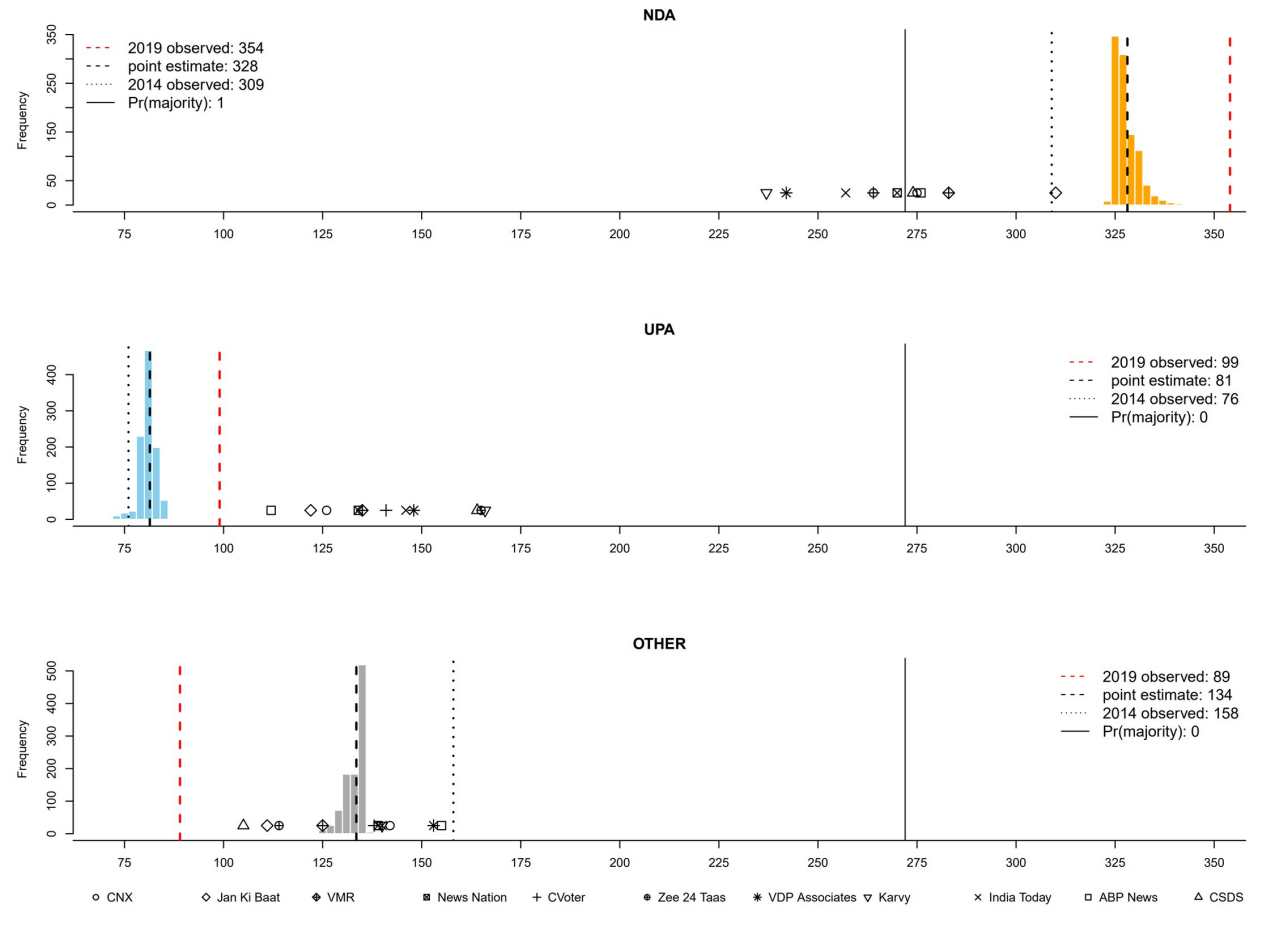
Seats projections at 5 weeks till the end of voting. *Pr*(majority) indicates the probability that a given party obtains an outright majority—272 seats or more—in the Lok Sabha.
