# Supplementary material for: Polling India via regression and post-stratification of non-probability online samples
Source: PLoS One. 2021 Nov 29;16(11):e0260092. doi: 10.1371/journal.pone.0260092 (PMC8629219; doi:10.1371/journal.pone.0260092)
Supplement: S1 Appendix — (PDF) [file pone.0260092.s001.pdf]

Polling India via Regression and Post-Stratification of Non-Probability Online Samples: **S1 Appendix**

Roberto Cerina[1,*], Raymond Duch[2],

1 Data Analytics and Digitalisation, Maastricht University, Maastricht, Netherlands

2 Nuffield College, University of Oxford, Oxford, U.K.

A1 Party Allocation to Alliance

Table S1 displays our allocation of parties to alliances; this was constructed based on the results found on `Wikipedia`, namely pages `https://en.wikipedia.org/wiki/List_of_National_Democratic_Alliance_members` and `https://en.wikipedia.org/wiki/List_of_United_Progressive_Alliance_members`, as well as unfolding news stories during the campaign. Parties not mentioned in the table were allocated to the 'Other' category. Note that there are several other parties which belong to these alliances, many of which have been created uniquely for the 2019 election. Where these residual parties have no chance of being competitive in any district, they are ignored, as they will not impact our analysis.

Party	Alliance
ajsu party	NDA
all india anna dravida munnetra kazhagam	NDA
all india n.r. congress	NDA
apna dal (soneylal)	NDA
asom gana parishad	NDA
bharath dharma jana sena	NDA
bharatiya janata party	NDA
bodoland peoples front	NDA
desiya murpokku dravida kazhagam	NDA
indigenous people's front of tripura	NDA
janata dal (united)	NDA
lok jan shakti party	NDA
manipur people's party	NDA
mizo national front	NDA
naga peoples front	NDA
nationalist democratic progressive party	NDA
national people's party	NDA
pattali makkal katchi	NDA
rashtriya loktantrik party	NDA
rashtriya samaj paksha	NDA
republican party of india (a)	NDA
shiromani akali dal	NDA
shivsena	NDA
sikkim democratic front	NDA
tamil maanila congress (moopanar)	NDA
united democratic party	NDA
bahujan vikas aaghadi	UPA
communist party of india	UPA
communist party of india (marxist)	UPA
communist party of india (marxist-leninist) (liberation)	UPA
dravida munnetra kazhagam	UPA
hindustani awam morcha (secular)	UPA
indian national congress	UPA
indian union muslim league	UPA
jammu & kashmir national conference	UPA
jan adhikar party	UPA
janata dal (secular)	UPA
jharkhand mukti morcha	UPA
jharkhand vikas morcha (prajatantrik)	UPA
karnataka pragnyavantha janatha party	UPA
kerala congress (m)	UPA
nationalist congress party	UPA
peace party	UPA
rashtriya janata dal	UPA
rashtriya lok dal	UPA
rashtriya lok samta party	UPA
revolutionary socialist party	UPA
swabhimani paksha	UPA
telangana jana samithi	UPA
telugu desam	UPA
viduthalai chiruthaigal katchi	UPA
vikassheel insaan party	UPA

Table S1. Our allocation of parties to alliances.

A2 Micro-Data for Integration

12

Source	Source_ID	States	Gender	Family_Role	Age_cat	Marital_Status	Rurality	Religion	Literacy	Education_level	Income_level	Jati	Zones	2014 Turnout	2014 Alliance_Vote
IHDS	1	(01) Jammu & Kashmir 01	(1) Male 1	(01) Head 1	(54-64]	(1) Married 1	(0) rural 0	(2) Muslim 2	(0) No 0	(01) No Formal Edu 01	(03) R 90,000 - R 1,19,999 03	(3) Other Backward Castes (OBC) 3	(01) North 01	NA	NA
IHDS	2	(01) Jammu & Kashmir 01	(2) Female 2	(02) Wife/Husband 2	(44-54]	(1) Married 1	(0) rural 0	(2) Muslim 2	(0) No 0	(01) No Formal Edu 01	(03) R 90,000 - R 1,19,999 03	(3) Other Backward Castes (OBC) 3	(01) North 01	NA	NA
IHDS	3	(01) Jammu & Kashmir 01	(1) Male 1	(03) Son/Daughter 3	(17-24]	(2) Unmarried 2	(0) rural 0	(2) Muslim 2	(1) Yes 1	(03) Middle or Secondary 03	(03) R 90,000 - R 1,19,999 03	(3) Other Backward Castes (OBC) 3	(01) North 01	NA	NA
IHDS	4	(01) Jammu & Kashmir 01	(1) Male 1	(03) Son/Daughter 3	(24-34]	(1) Married 1	(0) rural 0	(2) Muslim 2	(1) Yes 1	(03) Middle or Secondary 03	(03) R 90,000 - R 1,19,999 03	(3) Other Backward Castes (OBC) 3	(01) North 01	NA	NA
IHDS	5	(01) Jammu & Kashmir 01	(2) Female 2	(04) Child-in-Law 4	(24-34]	(1) Married 1	(0) rural 0	(2) Muslim 2	(1) Yes 1	(03) Middle or Secondary 03	(03) R 90,000 - R 1,19,999 03	(3) Other Backward Castes (OBC) 3	(01) North 01	NA	NA
⋮	⋮	⋮	⋮	⋮	⋮	⋮	⋮	⋮	⋮		⋮	⋮	⋮	⋮	⋮
IHDS	135980	(34) Pondicherry 34	(2) Female 2	(02) Wife/Husband 2	(44-54]	(1) Married 1	(1) urban 1	(1) Hindu 1	(1) Yes 1	(03) Middle or Secondary 03	(03) R 90,000 - R 1,19,999 03	(3) Other Backward Castes (OBC) 3	(06) Southern 06	NA	NA
IHDS	135981	(34) Pondicherry 34	(1) Male 1	(03) Son/Daughter 3	(24-34]	(2) Unmarried 2	(1) urban 1	(1) Hindu 1	(1) Yes 1	(05) Some Graduate or Higher 05	(03) R 90,000 - R 1,19,999 03	(3) Other Backward Castes (OBC) 3	(06) Southern 06	NA	NA
IHDS	135982	(34) Pondicherry 34	(1) Male 1	(03) Son/Daughter 3	(24-34]	(2) Unmarried 2	(1) urban 1	(1) Hindu 1	(1) Yes 1	(05) Some Graduate or Higher 05	(03) R 90,000 - R 1,19,999 03	(3) Other Backward Castes (OBC) 3	(06) Southern 06	NA	NA
IHDS	135983	(34) Pondicherry 34	(1) Male 1	(01) Head 1	(44-54]	(1) Married 1	(1) urban 1	(1) Hindu 1	(1) Yes 1	(03) Middle or Secondary 03	(03) R 90,000 - R 1,19,999 03	(3) Other Backward Castes (OBC) 3	(06) Southern 06	NA	NA
IHDS	135984	(34) Pondicherry 34	(2) Female 2	(02) Wife/Husband 2	(34-44]	(1) Married 1	(1) urban 1	(1) Hindu 1	(1) Yes 1	(03) Middle or Secondary 03	(03) R 90,000 - R 1,19,999 03	(3) Other Backward Castes (OBC) 3	(06) Southern 06	NA	NA
Census	1	(28) Andhra Pradesh 28	(1) Male 1	NA	(44-54]	NA	(0) rural 0	NA	NA	(01) No Formal Edu 01	NA	NA	(06) Southern 06	NA	NA
Census	2	(29) Karnataka 29	(1) Male 1	NA	(64-100]	NA	(0) rural 0	NA	NA	(01) No Formal Edu 01	NA	NA	(06) Southern 06	NA	NA
Census	3	(19) West Bengal 19	(1) Male 1	NA	(34-44]	NA	(0) rural 0	NA	NA	(01) No Formal Edu 01	NA	NA	(04) Eastern 04	NA	NA
Census	4	(08) Rajasthan 08	(1) Male 1	NA	(44-54]	NA	(1) urban 1	NA	NA	(05) Some Graduate or Higher 05	NA	NA	(01) North 01	NA	NA
Census	5	(33) Tamil Nadu 33	(1) Male 1	NA	(34-44]	NA	(0) rural 0	NA	NA	(04) Higher Secondary 04	NA	NA	(06) Southern 06	NA	NA
⋮	⋮	⋮	⋮	⋮	⋮	⋮	⋮	⋮	⋮		⋮	⋮	⋮	⋮	⋮
Census	3800209	(19) West Bengal 19	(2) Female 2	NA	(54-64]	NA	(1) urban 1	NA	NA	(03) Middle or Secondary 03	NA	NA	(04) Eastern 04	NA	NA
Census	3800210	(06) Haryana 06	(2) Female 2	NA	(54-64]	NA	(1) urban 1	NA	NA	(02) Primary or Lower 02	NA	NA	(01) North 01	NA	NA
Census	3800211	(01) Jammu & Kashmir 01	(1) Male 1	NA	(17-24]	NA	(0) rural 0	NA	NA	(02) Primary or Lower 02	NA	NA	(01) North 01	NA	NA
Census	3800212	(23) Madhya Pradesh 23	(1) Male 1	NA	(24-34]	NA	(1) urban 1	NA	NA	(02) Primary or Lower 02	NA	NA	(02) North-Central 02	NA	NA
Census	3800213	(20) Jharkhand 20	(1) Male 1	NA	(24-34]	NA	(1) urban 1	NA	NA	(03) Middle or Secondary 03	NA	NA	(04) Eastern 04	NA	NA
NES	1	(28) Andhra Pradesh 28	(1) Male 1	NA	(54-64]	NA	NA	(1) Hindu 1	NA	(02) Primary or Lower 02	(01) Less than R 60,000 01	(2) Forward/General (except Brahmin) 2	(06) Southern 06	1	NDA
NES	2	(28) Andhra Pradesh 28	(2) Female 2	NA	(44-54]	NA	NA	(1) Hindu 1	NA	(01) No Formal Edu 01	(01) Less than R 60,000 01	(5) Scheduled Tribes (ST) 5	(06) Southern 06	1	OTHER
NES	3	(28) Andhra Pradesh 28	(2) Female 2	NA	(34-44]	NA	NA	(1) Hindu 1	NA	(01) No Formal Edu 01	(01) Less than R 60,000 01	(2) Forward/General (except Brahmin) 2	(06) Southern 06	1	NDA
NES	4	(28) Andhra Pradesh 28	(2) Female 2	NA	(64-100]	NA	NA	(1) Hindu 1	NA	(01) No Formal Edu 01	(01) Less than R 60,000 01	(2) Forward/General (except Brahmin) 2	(06) Southern 06	1	NDA
NES	5	(28) Andhra Pradesh 28	(1) Male 1	NA	(64-100]	NA	NA	(1) Hindu 1	NA	(01) No Formal Edu 01	(01) Less than R 60,000 01	(2) Forward/General (except Brahmin) 2	(06) Southern 06	1	NDA
⋮	⋮	⋮	⋮	⋮	⋮	⋮	⋮	⋮	⋮		⋮	⋮	⋮	⋮	⋮
NES	22291	(28) Andhra Pradesh 28	(2) Female 2	NA	(34-44]	NA	NA	(3) Christian 3	NA	(01) No Formal Edu 01	(01) Less than R 60,000 01	(4) Scheduled Castes (SC) 4	(06) Southern 06	1	OTHER
NES	22292	(28) Andhra Pradesh 28	(2) Female 2	NA	(24-34]	NA	NA	(4) Sikh 4	NA	(01) No Formal Edu 01	(01) Less than R 60,000 01	NA	(06) Southern 06	1	OTHER
NES	22293	(28) Andhra Pradesh 28	(2) Female 2	NA	(34-44]	NA	NA	(4) Sikh 4	NA	(01) No Formal Edu 01	(01) Less than R 60,000 01	(3) Other Backward Castes (OBC) 3	(06) Southern 06	1	UPA
NES	22294	(28) Andhra Pradesh 28	(1) Male 1	NA	(17-24]	NA	NA	(7) Other 7	NA	(01) No Formal Edu 01	(01) Less than R 60,000 01	NA	(06) Southern 06	1	NA
NES	22295	(28) Andhra Pradesh 28	(2) Female 2	NA	(17-24]	NA	NA	(7) Other 7	NA	(01) No Formal Edu 01	(01) Less than R 60,000 01	(3) Other Backward Castes (OBC) 3	(06) Southern 06	1	NDA

Table S2. Subset of the stacked dataset derived from appending the NES and the Census micro-data sample to the IHDS. Missing values are indicated as NA

A3 Pre- and Post-Raking Stratification Frame

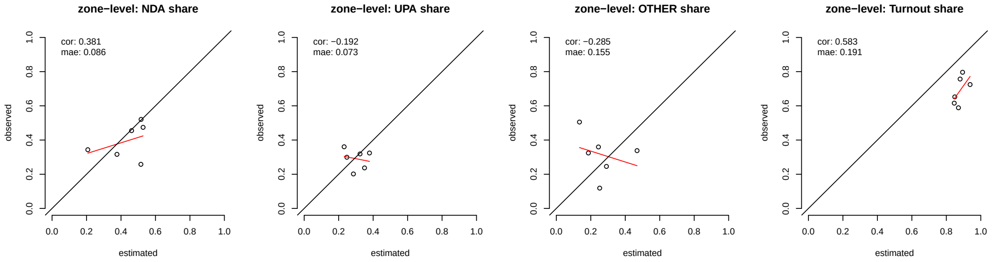

Fig S1. Post-imputation, pre-raking plot of voting distribution implied by the stratification frame at the Zone level.

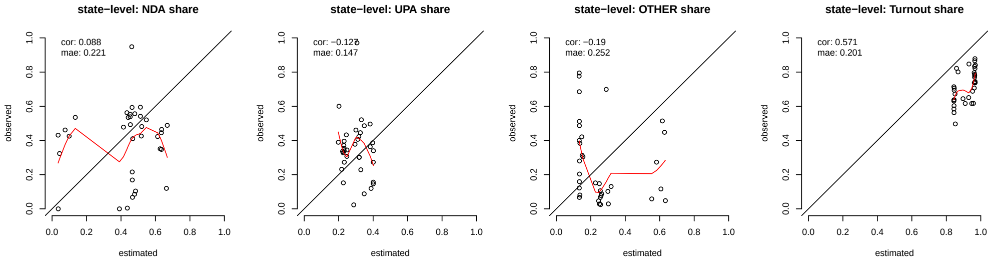

Fig S2. Post-imputation, pre-raking plot of voting distribution implied by the stratification frame at the State level.

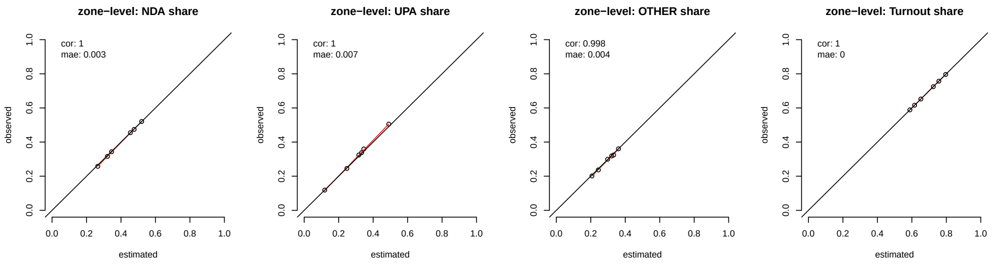

Fig S3. Post-imputation, post-raking plot of voting distribution implied by the stratification frame at the Zone level.

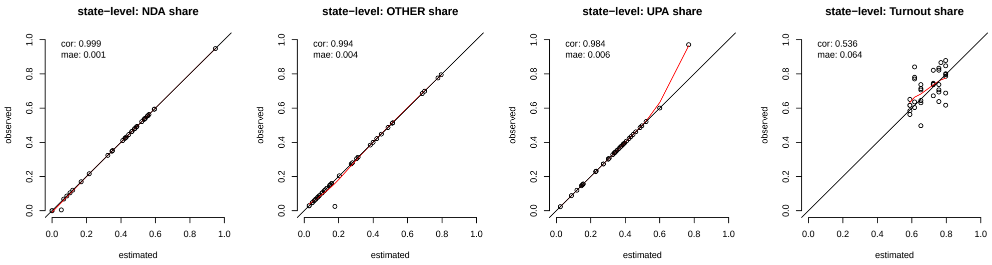

Fig S4. Post-imputation, post-raking plot of voting distribution implied by the stratification frame at the State level.

A4 Convenience Samples

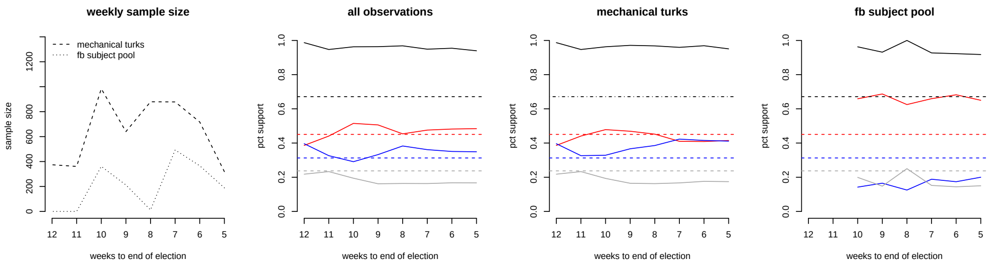

Fig S5. A graphical summary of the samples' evolution over the course of the collection period. The leftmost panel presents the sample size over-time, disaggregated by mode; the three plots to its right show % likely voters and alliance support over time (color coding: black = turnout; red = NDA; blue = UPA; gray = OTHER); we see how raw voting behaviour looks on the aggregate, as well as by mode.

Variable	Convenience Sample	2014 NES
Religion	0.16	
H.Inc.	0.56	
Gender	0.02	
Education		0.29
Age_cat	0.34	0.58
Caste	0.26	0.43
2014 Vote Choice	0.17	0.33
2014 Turnout	0.29	0.05
2019 Vote Choice	0.18	
2019 Turnout	0.20	

Table S3. Out-of-bag error derived from the `missRanger` multiple imputation procedure, by variables and training sample. The OOB score ranges $[0, 1]$, indicating the proportion of miss-classified OOB observations.

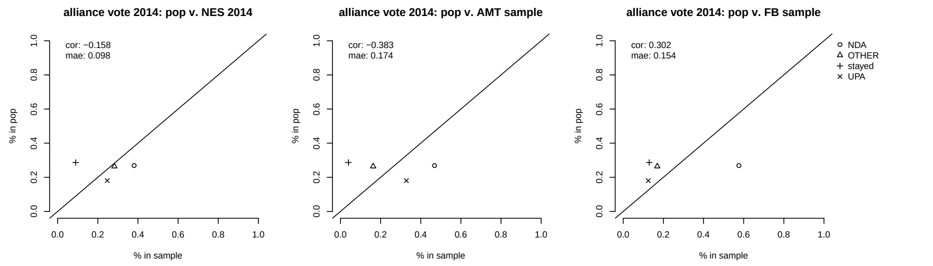

Fig S6. Micro-data samples compared with modeled population: 2014 Alliance Vote.

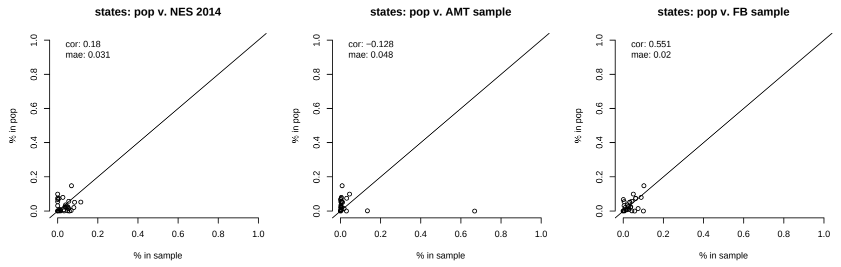

Fig S7. Micro-data samples compared with modeled population: States.

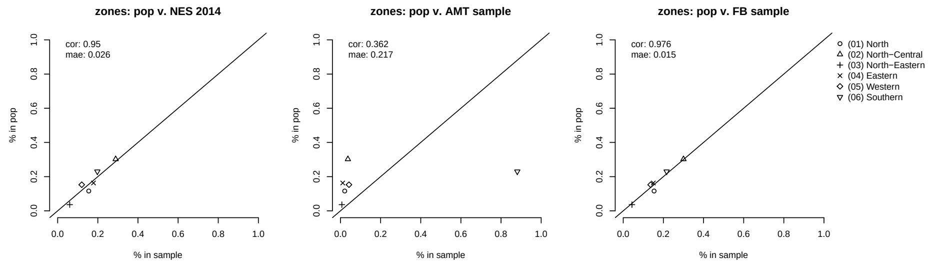

Fig S8. Micro-data samples compared with modeled population: Zones.

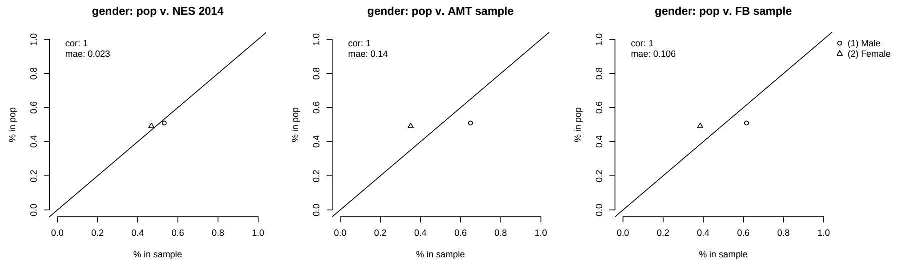

Fig S9. Micro-data samples compared with modeled population: Gender.

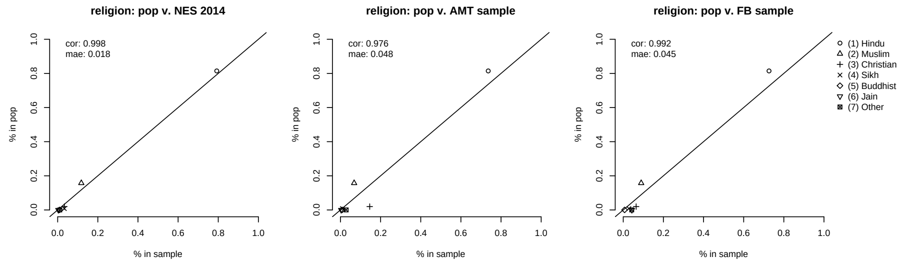

Fig S10. Micro-data samples compared with modeled population: Religion.

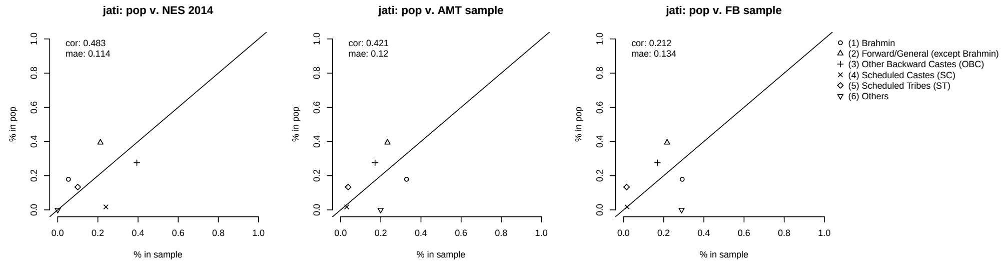

Fig S11. Micro-data samples compared with modeled population: Caste.

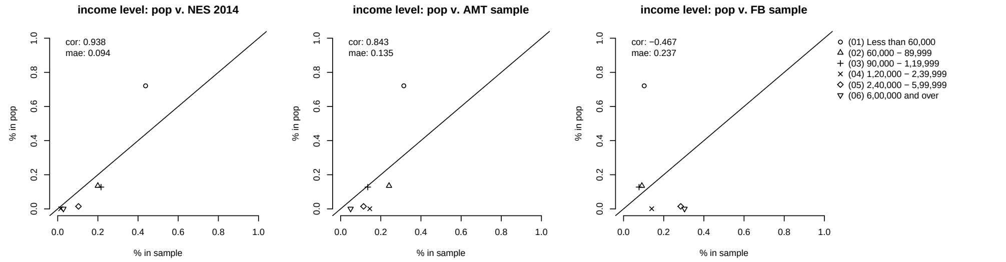

Fig S12. Micro-data samples compared with modeled population: Yearly Household Income.

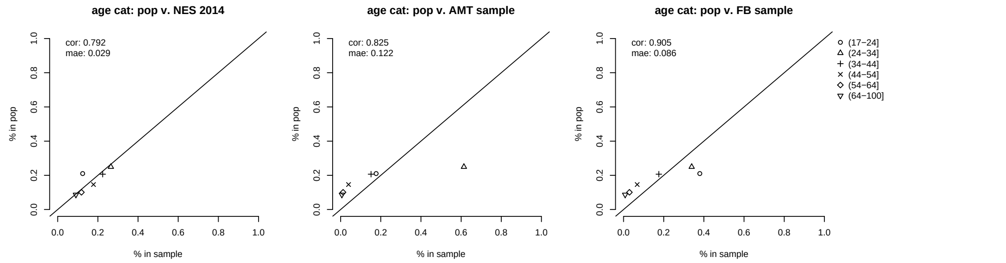

Fig S13. Micro-data samples compared with modeled population: Age Groups.

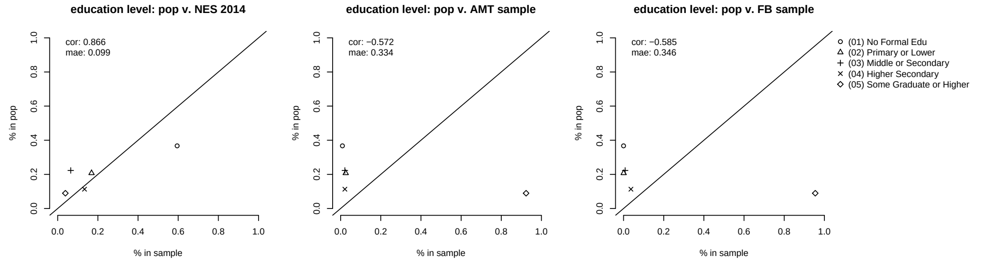

Fig S14. Micro-data samples compared with modeled population: Educational Attainment.

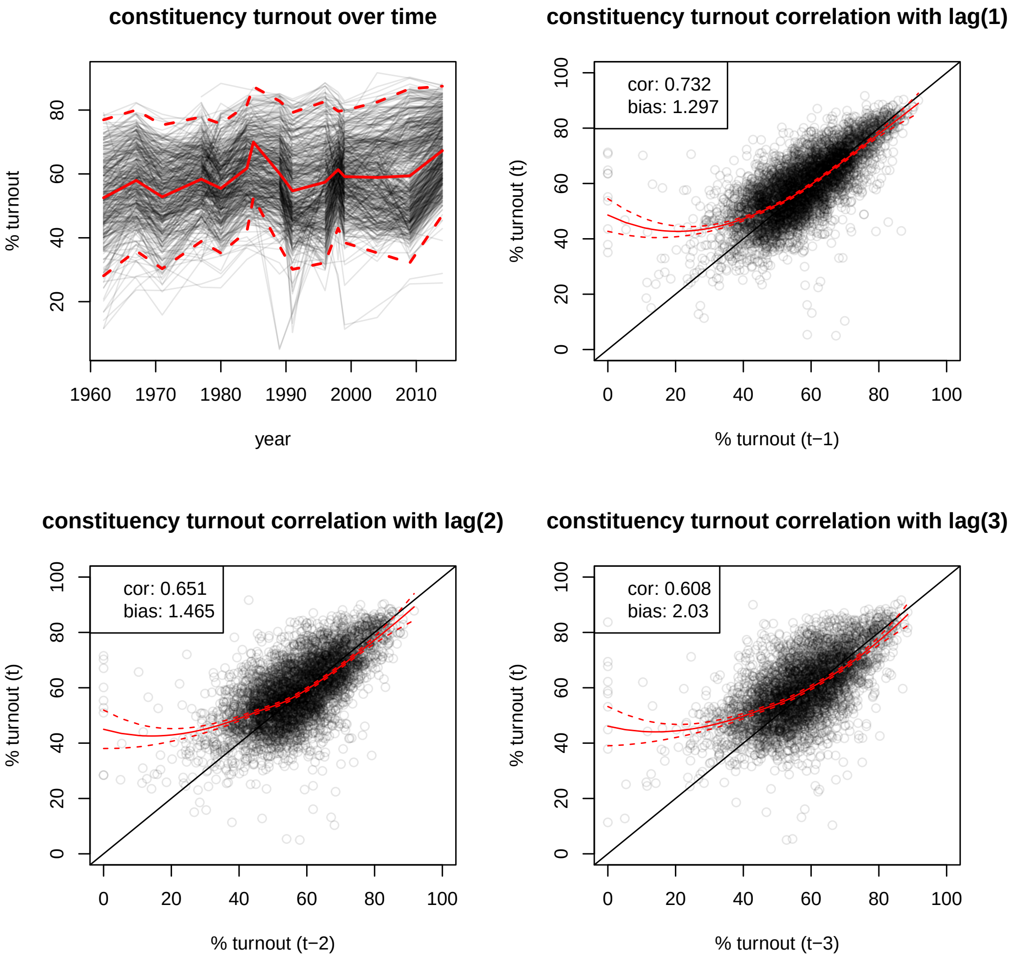

Fig S15. Constituency-level turnout across time.

A5 Modeling Behaviour

15

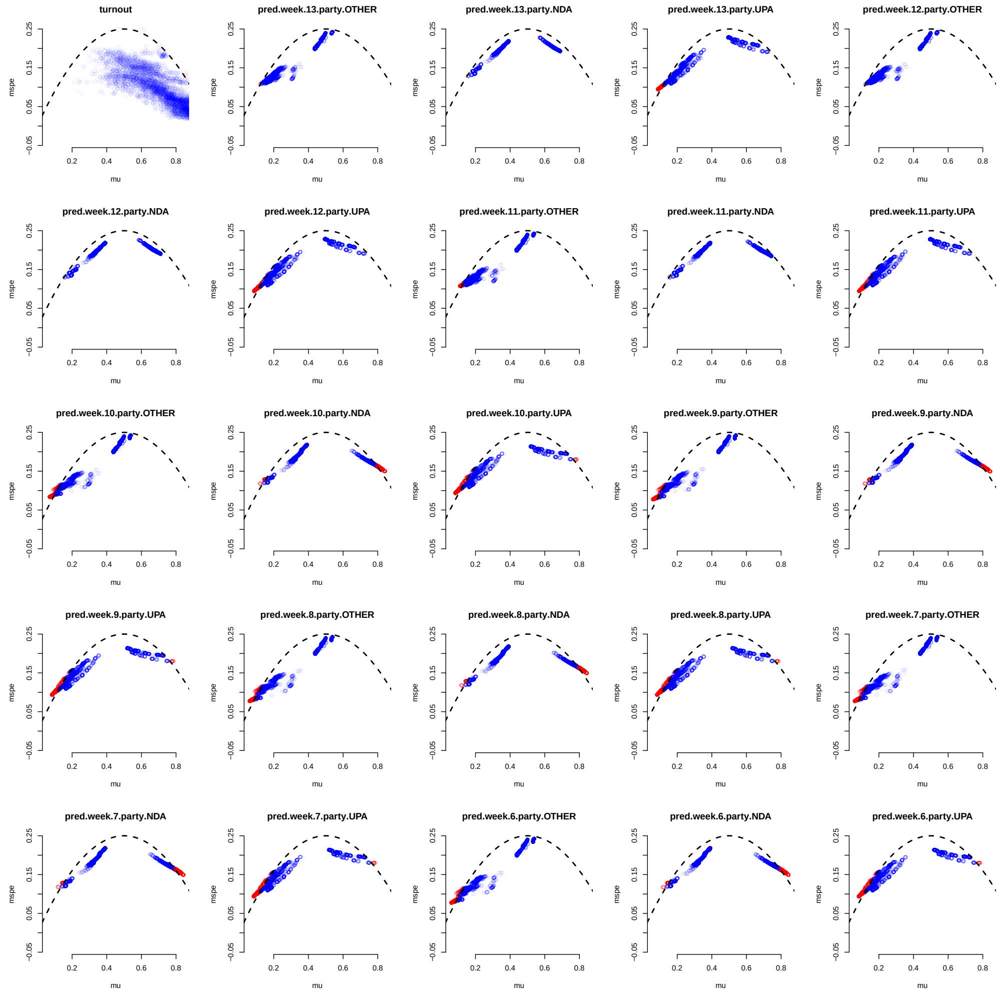

Fig S16. Predicted means and variances, against the variance limits required for conformity with the Beta distribution; red dots indicate non-conformity, and the $\mu(1-\mu)$ limit is shown by the dotted line.

Alliance	% Vote	% Vote Error	U-Swing Pred.	Raw Seats Error
NDA	50.8	-5.8	388	-34
UPA	32.4	-1.1	26	73
OTHER	16.8	6.9	129	-40

Table S4. Predictions derived from the raw tallies of alliance votes, conditional on raw turnout, from the online sample of Mechanical Turks. Seats predictions are derived according to a Uniform Swing model.

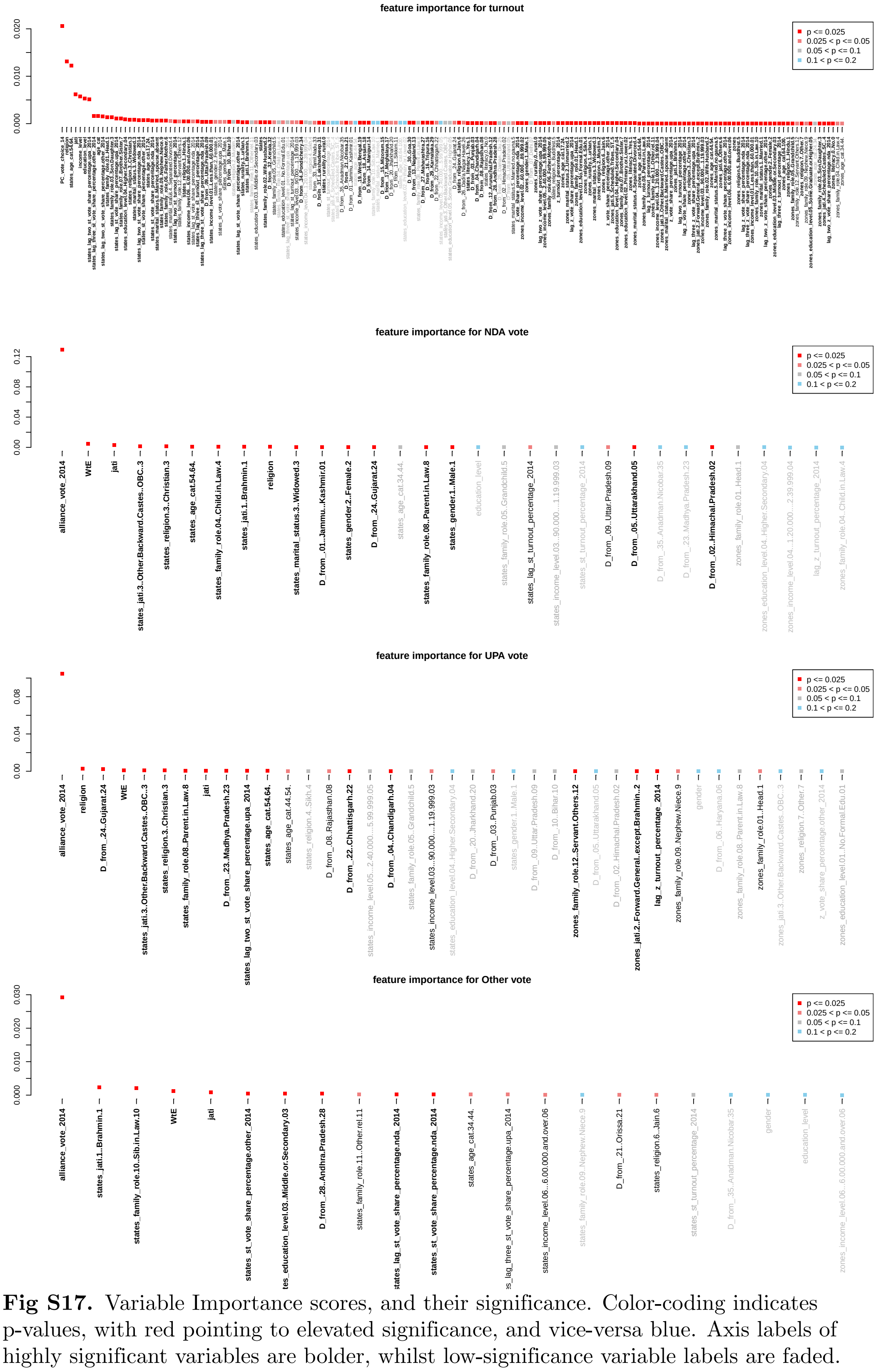

Fig S17. Variable Importance scores, and their significance. Color-coding indicates p-values, with red pointing to elevated significance, and vice-versa blue. Axis labels of highly significant variables are bolder, whilst low-significance variable labels are faded. State-level features are prefixed by 'states', whilst zone-level variables are prefixed by 'zone'; no prefix is attached to individual-level variables. Variables prefixed by 'D' are centroid distances from to given state.

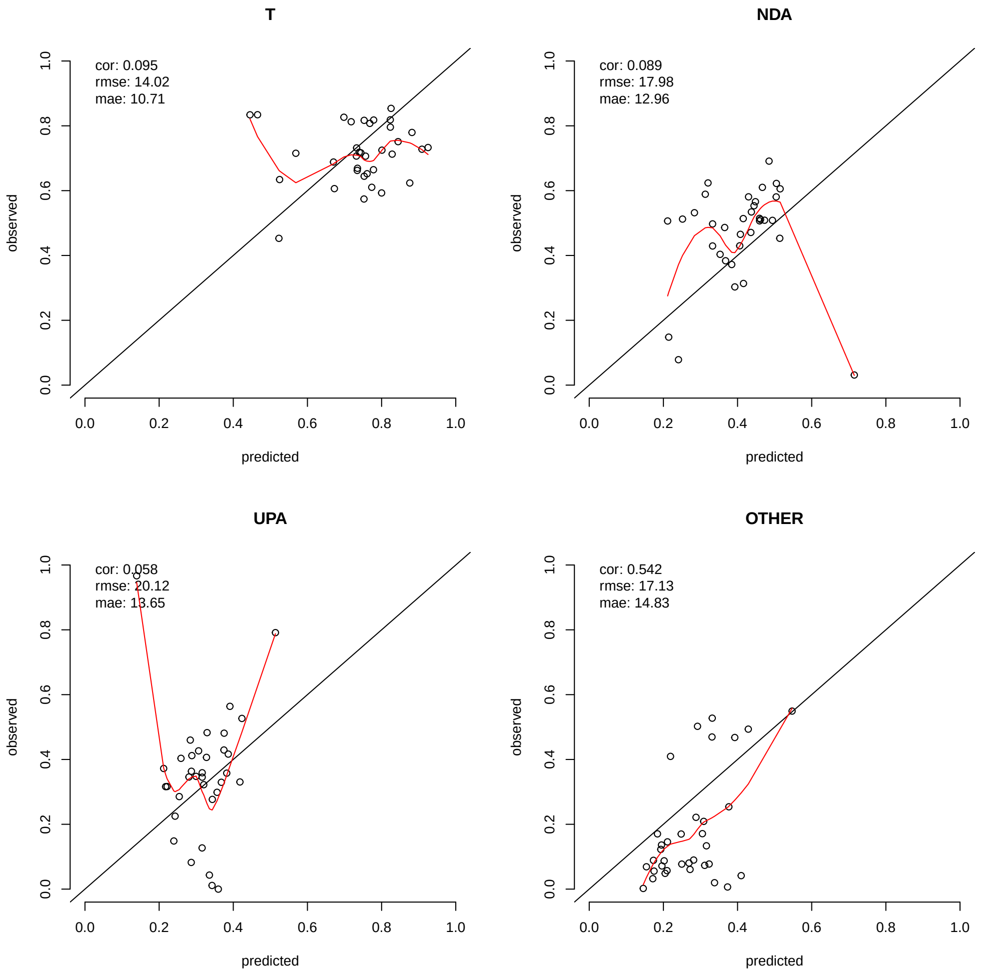

Fig S18. State-level predictions v. observed state-level 2019 behaviour.

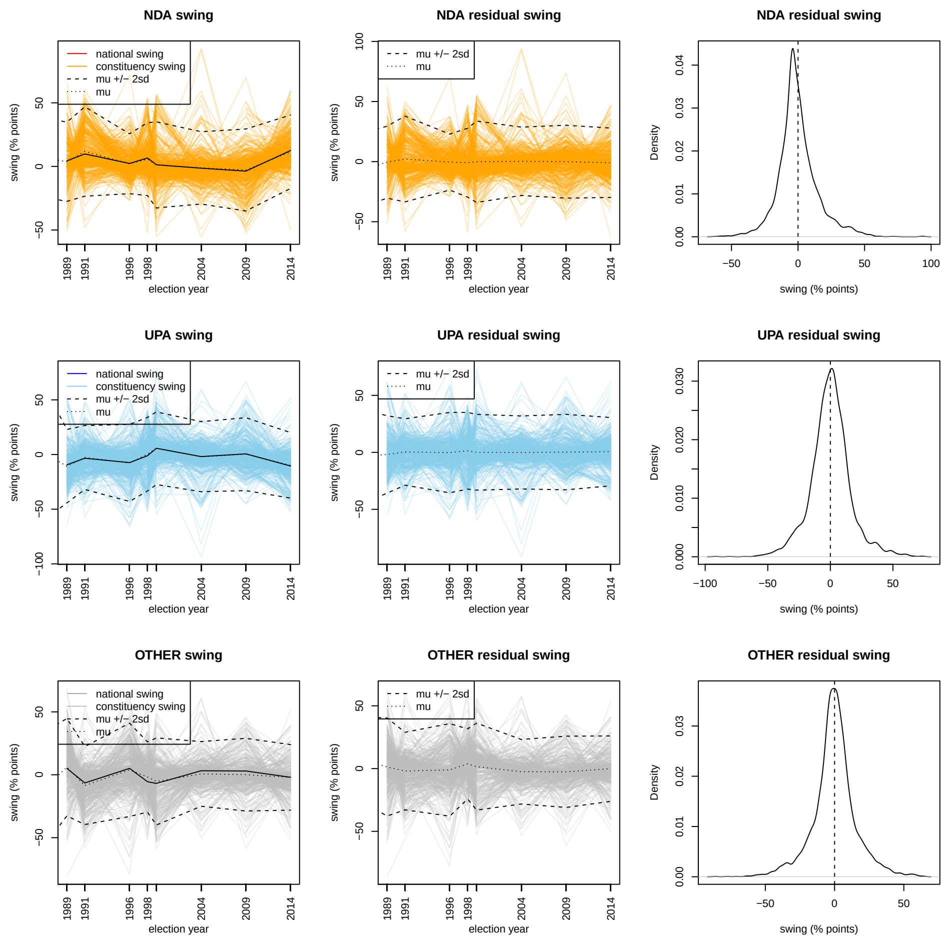

Fig S19. Analysis of constituency-level swing since 1989.

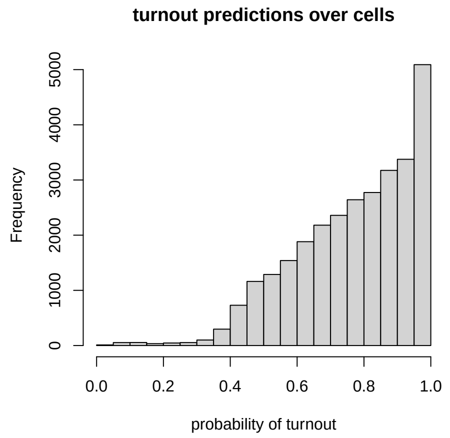

Fig S20. Predicted distribution of turnout across cells in the stratification frame.

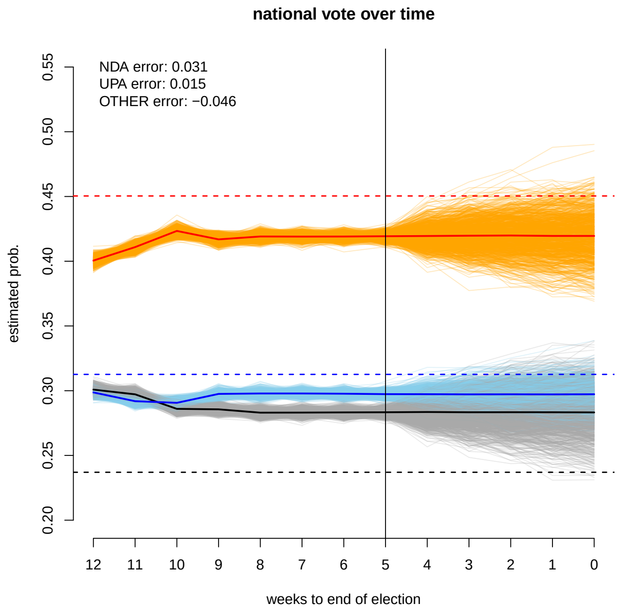

Fig S21. National-level predicted vote-share by alliance for a model which only uses vote-choice from the convenience sample, and does not account for turnout weights.
