# Supplementary material for: Polling India via regression and post-stratification of non-probability online samples
Source: PLoS One. 2021 Nov 29;16(11):e0260092. doi: 10.1371/journal.pone.0260092 (PMC8629219; doi:10.1371/journal.pone.0260092)
Supplement: Attachment — Submitted filename: PLOS-One Response to Reviewer Submission.pdf [file pone.0260092.s002.pdf]

Please submit your revised manuscript by July 10, 2021.

Additional Editor Comments:

1. GENERAL: Be very clear about what your model can predict, and the limitations;
2. In addition to the comments from the two reviewers, I was interested in knowing how will your model predict -- state elections in Tamil Nadu, Kerala, Rajasthan -- these are states that are known to change the ruling party every time it goes for elections;

   Response:

   We thank the Editor for the helpful comments and suggested revisions, which we have tried to fully satisfy to the best of our ability. The limitations of the approach highlighted by the reviewers have now been addressed in detail.

   With respect to the potential performance of our proposed method in Tamil Nadu, Kerala and Rajasthan, it is difficult to say a-priori whether the method will perform well, given that it has not yet been applied at the state-level. Based on what we have learned from this manuscript, Tamil Nadu and Kerala should be two populations which are very well suited for model-based polling via online samples, and especially via the study of Amazon Mechanical Turk workers. In that sense, as these populations are over-represented in online convenience samples, we can expect the strategy we outlined to be able to learn complex opinion and behaviours from moderately sized samples, and hence be a success. With respect to performance in Rajasthan, though we have no state-specific insight to make a specific claim, we believe our strategy to be founded on robust principles, and hence be applicable to this state. We aim to report on performance at deeper levels of analysis, such as the state and constituency level, in future research.

Reviewer #1:

This paper proposes and implements a method for using convenience online polling data (Facebook and Amazon's Mechanical Turk) to predict election outcomes in India. A simplified version of the steps of that process are the following: 1) use Indian census data to estimate the fraction of individuals in the population with particular combinations of characteristics; 2) generate a simulated micro-data sample based on that; 3) use the Indian National Election Survey and Indian Human Development Survey to impute characteristics for observations in that simulated microdata, such as past turnout; 4) calculate weights for the observations in the microdata so that statistics generated from that data will match aggregate statistics such as turnout; 5) use online polling data (from Facebook and Amazon's Mechanical Turk) from 2019 to model the relationship between individual-level characteristics and voting choices; 6) use data from the 2014 INES to model the relationship between individual-level characteristics and turnout; and 7) take that estimated relationship to the microdata to estimate turnout and voting decisions. The authors published predictions based on this methodology prior to the 2019 elections and these were more successful than public polls of the results. This suggests that this process for combining data sources can be used to make successful all-India election predictions.

Main Comments:

1. This paper addresses a difficult problem for which there are no perfect solutions – it is challenging to predict election outcomes in contexts where true random sampling is not possible. However, there are a number of challenges for the voter turnout modeling in the paper. The 2019 online polling data on intended turnout cannot be used to create a good turnout model since 97% of the sample reports that they will turn out to vote. The authors instead use the 2014 INES data, but even that is not very helpful for building a turnout model since nearly everyone reports having turned out to vote in that survey. As a result, the turnout predictions are poor: for example, looking at the state-level predictions of turnout in the upper right

figure of figure S17, the correlation between predicted and actual turnout is low and would be basically zero with one state excluded.

I do not think that the authors need to change this aspect of the paper in a revision – one of the strengths of the paper is that the authors made public predictions prior to the elections based on this model and then validated them against the actual election results. Instead, I think the authors should revise the paper to describe these weaknesses more clearly in the paper. As I see it, there are two points to highlight. First, they are using 2014 data to model 2019 turnout. As a result, their method would struggle in elections with differential turnout patterns across cycles. The authors should be clear about this point.

Second, it is more accurate to describe their method as based almost entirely on estimating voter preferences rather than about turnout; they just don't have data that would allow them to accurately predict turnout. That is fine as a limitation of this particular method, but it should be made clear to readers that they are really just trying to peg overall turnout to 2014 and then model voter preferences as a function of observable characteristics.

I. Response

The Reviewer highlights important limitations of our approach, especially as it relates to our cell-level turnout model. In particular, the Reviewer challenges us to a) be more explicit as to the limitations and consequences of using past turnout data from the last-election NES to train our cell-level turnout model; b) to be explicit about how much the turnout model is ultimately affecting vote-share and seat estimates.

We have thus modified the section 4.1 `Turnout Training Data' to include a more comprehensive justification for the use of 2014 NES training data for turnout, which more explicitly deals with the limitations of this approach. In particular, we highlight reasonably high year-on-year correlation in turnout at some deep-cell levels, and highlight comparable empirical research which has found success with this method. We include a brief comment in Footnote 22 in this section that argues for incorporating turnout probabilities into these types of forecasting exercises even when the estimated turnout probabilities are less than perfect.

The Reviewer's comments further allow us to extend our theory about hyper-parameter tuning for probability machines under highly unbalanced outcomes; a revised account of hyper-parameter tuning for our turnout model is available in section 5, under the paragraph `Hyper-Parameter Tuning'. The models have been re-fit to conform to this new, more sensible training strategy, and hence the results present some minor differences (especially at the deep-cell-level) from the previous submission.

We have further added to the appendix the results of a no-turnout model, which show clearly that modeling turnout reduces absolute error on the vote-share estimates by close to 2 percentage points over the three parties, worsening in particular the predictions about the UPA and Other alliances.

2. The authors are transparent in noting many of the limitations of their approach. I thought that there were a number of places where the authors need to more heavily caveat some weakness of their approach and how these weaknesses could cause their method to yield misleading estimates in other election cycles. This will also help readers who are interested in using similar methodologies to build future models of Indian elections.

a. The key reason that the method of authors was better at predicting election outcomes was that they were more optimistic about BJP performance and pessimistic about UPA performance than the public pollsters. It was not clear to me whether this is because of particularly strength of their methodology or because online samples happen to have unobservable characteristics that predispose them towards BJP (and against UPA) beyond that would be expected by observable characteristics. It seems like that may be true (page 21), and

as a result, it may not be that the method is generally strong, but that in an election where UPA underperforms polls/BJP overperforms, this bias happened to push them in the right direction.

II. Response:

This is a reasonable conjecture on the part of the Reviewer. But of course the forecasting method that we lay out has considerable value added above and beyond the results from our convenience samples which are BJP biased. By simply ignoring this value added component and relying entirely on the convenience sample for a forecast we get a rough read of whether we were simply lucky here. Accordingly, we have produced vote-share and Uniform Swing projections derived from the `raw' online convenience sample. If the Reviewer's assertion is correct, we would expect little to no difference in vote-share and seat predictions performance; to the contrary, we see a weaker national vote-share predictions (worth around 2% larger error per alliance, on average). Due to the non-linear uniform-swing effects, the error on the seat predictions using the raw vote-shares is far worse, as the over-estimation of the NDA vote results in a dramatic drop in UPA seats (error = 73 seats), and the NDA-UPA lead is over-estimated by over 100 seats. This analysis provides convincing evidence that our methodology is adding value to the raw data, and that the success of our predictions is not merely due to selection effects into the online sample. We point this out in Section 6, in a new paragraph on line 839.

b. In section 4.1, the "across the board correction" should be more accurately described as randomly selecting around a third of the sample to change the value of their reported turnout to zero. This is not a "correction" -- even under the very strong (and probably incorrect) assumption that misreporting past voter status is unrelated to characteristics of the individual, this is just introducing a bunch of noise into the predictions. The authors should be clearer about what this process is doing and the assumptions required for this to be asymptotically valid.

III. Response:

The reviewer is absolutely correct here, and pushes us to rethink our approach. We replace the uniform over-reporting correction for a simple, but more theoretically justifiable, re-weighting via raking. This is now explained at the end of section 4.1 `Turnout Training Data'. We are also transparent in admitting that this will not entirely address our over-reporting problems, nor can it truly provide us with new information - we cannot claim to be `correcting' the data. However, re-weighting does help with minimizing the shrinkage-error in national turnout predictions, which is exacerbated in unbalanced datasets. Though compared to our previous approach, this has the disadvantage of preserving a great deal of the upward bias in turnout predictions (around 11 points), it significantly improves the heterogeneity in cell-level predictions.

c. On pg 20, the paper describes it as an "oddity" that Christians are over-represented among Mechanical Turks as well as some other issues with that sample (e.g. more likely to support NDA/UPA). In all cases, it seemed clear that this was because Tamil Nadu and Kerala make up over 80% of the Mechanical Turk sample. For example, Kerala has the highest density of Christians in India. This could be mentioned there as the likely explanation. In general, given the geographical heterogeneity of India, it seemed like the Facebook data is more promising for all-India predictions in future election cycles.

IV. Response:

We thank the reviewer for pointing this out. The connection between Christian over-sampling and the geographical skew of our sample has been made clear in Section 4.2 `Severity of Non-Representation'.

d. On pg 13, the authors refer to deeper selection issues relating to the NES but do not describe them: I would appreciate it if the authors made these more explicit at that point or at least referred to points later in the paper that discuss this and how these may effect their overall results.

V. Response:

A footnote has been added describing the sort of unobservable selection we expect in a non-representative sample which favours likely voters, such as the INES.

Minor comments:

1. I wasn't able to access the data or find it on the Harvard dataverse that they report uploading it to, so they should update the links (or perhaps make it publicly searchable)

VI. Response:

Again, we thank the reviewer for pointing this out - this was a technical oversight on our part -- we did not hit the "publish" button on dataverse -- our apologies. The data has now been made available to the public at the following url: https://dataverse.harvard.edu/dataset.xhtml?persistentId=doi:10.7910/DVN/0RRVKJ

2. In section 3.2, the process for raking the NES data to the known 2014 election results at the state level was not clear – what variables were used when generating the raking weights? This should be described more carefully.

VII. Response:

A footnote has been added describing the way in which the raking procedure works, and which variables are used to re-weight the INES.

3. Rahul Gandhi's name is misspelled on page 5.

VIII. Response:

This has now been fixed.

4. On page 17, I did not understand why there were more voting intentions than there were users – is this a single IP address completing the survey multiple times? Please explain in more detail.

IX. Response:

The Reviewer is correct that we have multiple responses for each IP address. This has now been made clearer in the text.

5. In figure S5, there should be a legend added to explain the color coding used there.

X. Response:

A color-coding scheme has been added to the caption of the Figure.

Reviewer #2:

The paper proposes a method of data collection and analysis to project electoral outcome during campaign in the Indian context. The authors conduct repeated online polling of a non-representative sample of voters and use various census and survey datasets to project voting behavior for the entire population to estimate the aggregate turnout, vote shares and seat shares of the main coalitions for the 2019 general election in India. The method produces very good predictions for all the three measures, and therefore, may have the potential to be used more widely to get better early predictions of election outcomes. Below I describe my comments highlighting various issues with the paper.

Main Comments:

1. The first major issue with the paper is its writing. The authors have not managed to articulate their work very well. Consequently, I was left confused in many segments and had to make educated guesses about what they were doing. The LHS of equation (1), for example, is defined simply as "vote choice" of the Indian voters. From discussions several pages later it appears that it is a vector of vote shares of three coalitions. It is not mentioned what is "X" or the index "i" in equation (1) that the authors are summing over. The same equation appears again later in the paper, where some of the expressions are explained. Similarly, it is not well-explained what is being depicted in Figure 1. Specifically, what do the points in the graph represent? The paper needs significant rewriting to make the details clear.

I. Response:

We have addressed the unclear notation in equation 1 by providing a more detailed description of the variables in the equation as a footnote. With respect to Figure 1, we have added a clearer description of what the dots mean in the caption.

2. The main contribution of the work seems to be predicting the aggregate vote shares of the three coalitions (NDA, UPA and Others) quite well. The prediction is a combination of two exercises – continuous and real time opinion polls in two online platforms and projecting the reported behavior of this non-representative sample on the population. Since both exercises are "new" relative to the existing opinion polls in India (more on this in the next point), it would be good to know the relative importance of each exercise in ensuring a good prediction. Specifically, what would the vote share predictions be if the opinion polls are taken as they are, without adjusting for the non-representativeness of the underlying sample? This would tell us how much of additional improvement in prediction the authors achieve by employing the statistical technique.

II. Response:

This has been addressed in response to a similar comment brought up by the first Reviewer - vote shares, and seat-shares according to a Uniform Swing projections, from the raw opinion polls (no-modeling) are presented in Table S4 in the Appendix; a discussion comparing national vote-share estimates from modeled and un-modeled polls is now available in Section 6, under the paragraph `National Behaviour'; a direct comparison of the raw and modeled seat-share estimates has been added in Table 3. The modeling effort conclusively adds value in both vote-share and seat predictions.

3. In relation to the above point a discussion of a particular contextual detail may be important. There was a major incident that happened before Lok Sabha election in 2019 that became politically highly salient. On February 26, 2019, there was a terror attack on an army convoy in Kashmir, which escalated into a major tension between India and Pakistan, from which the incumbent government seemed to have electorally gained in the 2019 elections. The incident happened around 12 weeks prior to the start of the elections, exactly around the time Figure 3 depicts the vote shares. As evident from the Figure, the NDA's vote share rises sharply around that time, before stabilizing around week 9. This shift in behavior would not be picked up in the other opinion polls as most of them were conducted before the incident, as Table 3 shows. In that

sense, (repeated) online opinion polls have the advantage of picking up shocks to voter preferences that occur closer to elections that standard survey based opinion polls may not be able to capture. The authors should highlight this point. Moreover, if the shift in preference in favor of NDA was uniform across population groups, then the additional advantage of using the authors' statistical method may not be that high in this specific application. It is therefore important for them to demonstrate the marginal contribution of the method.

III. Response:

We thank the reviewer for highlighting this important piece of context for the 2019 election, which we feel further emphasizes the strength of our method: the spike in NDA vote share, which corresponds to the self-styling of the BJP as the `Watchmen', highlighting their focus on issues of National security, is well captured by our methodology. This has been highlighted in a new paragraph of Section 6.

With respect to the marginal contribution of the demographic adjustment resulting from our model efforts, we have addressed this in our earlier comment and also in response to Reviewer 1. As we point out in our response, the additional estimations and data presentations in Section 6, Table 3 and Appendix Table S4 explicitly show the added value of modeling in vote-share and seat estimation. We note however that there is independent value of modeling preferences beyond demographic re-weighting - namely being able to smooth party support over time enables borrowing of strength across time-points, hence providing more robust estimation of campaign effects. More broadly, we argue model-based polling is a good idea, and this paper provides evidence in favour of this conjecture.

4. The method to convert the vote shares to seat shares is rather crude, and in general, it would not work. My sense is that it worked well in this specific case because the shift in vote in favor of NDA in 2019 relative to 2014 was mostly uniform across constituencies, thanks to the general positive attitude in favor of the incumbent Prime Minister, who was the main face of NDA. I suggest the authors move the entire discussion on seat share to the appendix, unless they are able to propose a significantly better method.

IV. Response:

We thank the reviewer for their thoughtful comments here, though we respectfully disagree with their assertion regarding the lack of success with Uniform Swing in the Indian context. There are two main reasons to advocate for the use of Uniform Swing in the Indian context: one is empirical in nature, the other speaks to gains in transparency in what are otherwise opaque polling practices.

From an empirical perspective, uniform swing is supported by the historical election data, as shown by a swing analysis of the three alliance groupings (NDA, UPA and Other) which we have now included in the appendix (Fig. S19) and discussed in the `Seat Projections' paragraph of Section 6. Though this analysis is only valid for the specific alliance make-up of 2019 (i.e. the specific combination of parties which formed the alliances in 2019, which is different from previous years), there is strong evidence that the constituency-level swings are symmetrically distributed around the national swing. This is in line with long-standing research by Butler et al., who first provided evidence for uniform swing patterns in India.

From a polling-methodology perspective, advocating for, and using the, uniform swing to obtain seats projection is a clean, transparent and easily understandable model to obtain seat-level projections. Transparency is a quality that is sorely lacking in pre-election Indian polls, and in particular the steps which allow pollsters to go from National vote-shares to seats (the latter being the only quantities the majority of polls tend to publish) has been shown to be the most opaque (see https://blogs.lse.ac.uk/southasia/2016/06/10/polling-one-billion-measuring-public-opinion-in-the-worlds-largest-democracy/). To the extent that our paper attempts to set a new standard for `Polling

1 Billion', we advocate for the use of Uniform swing projections, until novel methodologies which have more empirical support from the data emerge.

5. The authors use NES 2014 to compute turnout probabilities. Given the really high reported turnout even in NES 2014, it seems there are other options. For example, the authors can use constituency level true turnout figures and regress them on constituency level demographic and other variables (available from SHRUG data: http://www.devdatalab.org/shrug) to get at how population characteristics map to turnout behavior.

XV. Response:

We thank the reviewer for pointing us toward this outstanding resource (SHRUG) which we will absolutely make use of in future iterations of this work. With respect to turnout modeling in our paper, we feel we have provided a robust response to criticisms with respect to the use of past-election high-quality survey data, which we underscore is a limitation of this study, but argue all-in-all is likely to provide the best possible approximation of cell-level turnout available in the India 2019 landscape.

We present two strong arguments for modeling turnout based on national election survey data from past elections: first turnout is relatively stable at the deep cell level which is indicated by the stability of turnout at constituency levels; and second, the existing literature on regression and post-stratification makes accurate predictions using this paradigm.

Finally we note that including our turnout probability estimation strategy in the modeling clearly produces better results than if we were to ignore turnout and only focus on voting intention (as shown by a comparison of Figure 3 with Figure S21 in the appendix). This in our minds justifies our approach as a net-positive contribution and is consistent with current practices in the literature. We finally highlight that even with a resource like SHRUG, we could only make ecological inference with respect to deep cell-level inference, whilst survey data allows us to make meso-level inference at the deep cell-level.
