# Supplementary material for: Polling India via regression and post-stratification of non-probability online samples
Source: PLoS One. 2021 Nov 29;16(11):e0260092. doi: 10.1371/journal.pone.0260092 (PMC8629219; doi:10.1371/journal.pone.0260092)
Supplement: Attachment — Submitted filename: Referee Reprt 2.pdf [file pone.0260092.s003.pdf]

Polling India via Regression and Post-Stratification of Non-Probability Online Samples

Roberto Cerina and Raymond Duch

Second Referee report

The authors have addressed some of the issued raised in my first review.

Regarding turnout prediction, the point is not whether the authors' analysis is informative, but whether there are better methods of doing so. Ceteris paribus, it is certainly better to use survey data than rely on ecological inference. However, there is significant over-reporting of turnout in NES. Importantly, the degree of over-reporting is likely to be different across population groups. Hence, it is not obvious that using survey data would give better prediction than my proposed method.

The other point I would make is with regard to the authors' defense in favor of their uniform swing assumption underlying the seat share projection analysis. The authors correctly point out that distribution of swings at the constituency level is typically Normally distributed around the national level swing. However, from that the authors imply: "We can expect some constituencies to go the opposite way of the national-level swing, and others to be swinging more heavily toward that trend. But these extremes will appear at roughly the same frequencies and hence cancel each other out." (page 38) This is not a correct argument. In first past the post system, extremes "cancelling each other out" need not happen. It depends on which specific constituencies get those extreme shocks. Generally speaking, how a given distribution of swing translates into seats depends critically on the assignment of the swing shocks to constituencies. The same shock may or may not change the winner's party depending on the previous win margin of the constituency. I would therefore request the authors to remove that discussion from the paper.
