# Supplementary material for: Polling India via regression and post-stratification of non-probability online samples
Source: PLoS One. 2021 Nov 29;16(11):e0260092. doi: 10.1371/journal.pone.0260092 (PMC8629219; doi:10.1371/journal.pone.0260092)
Supplement: Attachment — Submitted filename: Rebuttal Letter - Round 2.pdf [file pone.0260092.s004.pdf]

Rebuttal Letter for 2nd Round of Revisions for Paper: `Polling India via Regression and Post-Stratification of Non-Probability Online Samples'

Roberto Cerina and Raymond Duch

We thank the reviewers for their very helpful comments - we have implemented all of their suggestions into the paper. Areas where the new manuscript differs from the last revision are highlighted in yellow in the `Tracked Changes' version. We are grateful to the reviewers and the editor and feel the quality of the paper has significantly improved as a result of the revision process.

Reviewer 1:

Minor Comments:
- Footnote 12: "social trus " should be "social trust." The footnote should also come after the period rather than before.
This has been addressed - footnote 12 now spells ` social trust' .

- Footnote 22: this footnote should be placed after the period rather than before.
This has been addressed - footnote 22 is now placed after the period.

- Page 21: please include a reference to the figure numbers for the sample v population plots in the appendix so it is easier for the reader to find them.
This has been addressed - a new sentence including a specific reference and description for each of the pop. v. sample figures in the appendix is now present on page 22.

- Page 29: "advice fore the" should be "advice for the"
This has been addressed - now on page 30.

- Page 33: "attributable to the quality the online pre-election polling" should be "to the quality of the online pre-election polling". The sentence "put plainly, to what extent is our prediction more accurate as a result of modeling conditional on turnout ? and is the turnout modeling architecture justified ?" should be "put plainly, to what extent is our prediction more accurate as a result of modeling conditional on turnout, and to what extent is the turnout modeling architecture justified?"
This has been addressed - now on page 33 it reads as the reviewer suggests.

- Fig S21: "convenicence" should be "convenience"
This has been addressed - now on page 58 it reads as the reviewer suggests.

Reviewer 2:

The authors have addressed some of the issued raised in my first review.

Regarding turnout prediction, the point is not whether the authors' analysis is informative, but whether there are better methods of doing so. Ceteris paribus, it is certainly better to use survey data than rely on ecological inference. However, there is significant over-reporting of turnout in NES. Importantly, the degree of over-reporting is likely to be different across population groups. Hence, it is not obvious that using survey data would give better prediction than my proposed method.
We agree with the reviewer - we now have included a footnote on page 21, at the end of our discussion of turnout modeling, where we highlight the possibility of using SHRUG data to obtain unbiased predictions for turnout, and present a few ideas on how future research could include SHRUG data in our estimation strategy.

The other point I would make is with regard to the authors' defense in favor of their uniform swing assumption underlying the seat share projection analysis. The authors correctly point out that distribution of swings at the constituency level is typically Normally distributed around the national level swing. However, from that the authors imply: "We can expect some constituencies to go the opposite way of the national-level swing, and others to be swinging more heavily toward that trend. But these extremes will appear at roughly the same frequencies and hence cancel each other out." (page 38) This is not a correct argument. In first past the post system, extremes "cancelling each other out" need not happen. It depends on which specific constituencies get those extreme shocks. Generally speaking, how a given distribution of swing translates into seats depends critically on the assignment of the swing shocks to constituencies. The same shock may or may not change the winner's party depending on the previous win margin of the constituency. I would therefore request the authors to remove that discussion from the paper.
We agree with the reviewer that our phrasing of the discussion on swings canceling out is inaccurate - we have now removed this discussion point on page 38.
